# Surveillance of arthropod-borne viruses in Benin, West Africa 2020–2021: detection of dengue virus 3 in *Aedes aegypti* (Diptera: Culicidae)

**DOI:** 10.1186/s40779-022-00425-9

**Published:** 2022-11-14

**Authors:** Carine Tchibozo, Gildas Hounkanrin, Anges Yadouleton, Alexandra Bialonski, Eric Agboli, Renke Lühken, Jonas Schmidt-Chanasit, Hanna Jöst

**Affiliations:** 1 Laboratory of Viral Haemorrhagic Fevers and Arboviruses of Benin, 01BP 918, Cotonou, Benin; 2 Centre de Recherche Entomologique de Cotonou, 01BP 918, Cotonou, Benin; 3 Ecole Normale Supérieure de Natitingou, National University of Science, Technology, Engineering and Mathematics, Abomey, Benin; 4 Bernhard Nocht Institute for Tropical Medicine, WHO Collaborating Centre for Arbovirus and Haemorrhagic Fever Reference and Research, 20359 Hamburg, Germany; 5 University of Health and Allied Sciences, PMB 31, Ho, Ghana; 6 Faculty of Mathematics, Informatics and Natural Sciences, Universität Hamburg, 22609 Hamburg, Germany

**Keywords:** Dengue virus, Arbovirus, Mosquitoes, *Aedes aegypti*, Benin

Dear Editor,

Dengue virus (DENV, family *Flaviviridae*, genus *Flavivirus*) serotypes 1 to 4 (DENV-1, -2, -3, and -4) are responsible for more than 100 million infections per year worldwide. Symptoms of DENV infection can be diverse, reaching from an acute febrile illness to the more severe, sometimes fatal dengue haemorrhagic fever/dengue shock syndrome. After the primary infection, lifelong immunity against a specific serotype is built. However, secondary infections by heterologous serotypes increase the risk of severe disease. First epidemics in Africa date back to the nineteenth century, and since the 1960s laboratory-confirmed cases and outbreaks have been reported in many countries in sub-Saharan Africa [1]. All four serotypes circulate in Africa with DENV-1 and DENV-2 being described most frequently. Due to the lack of specific diagnostics, DENV infections are often misdiagnosed with diseases that share a similar clinical presentation, e.g., malaria, chikungunya, Zika, yellow fever, or typhoid fever. A valuable, non-invasive tool to detect the circulation of arthropod-borne viruses (arboviruses) is entomological surveillance. Monitoring arboviruses in field-collected mosquitoes can serve as a tool to guide the appropriate control measures prior to arbovirus epidemics. In addition, it allows the investigation of arbovirus evolution or adaptation, as well as the identification of new lineages, and provides data about vectors that are involved in the local arbovirus transmission.

In Africa, data about arbovirus distribution and prevalence mostly come from seroprevalence surveys or single case studies, i.e., travellers returning to countries outside of Africa. Reported cases are suggested to significantly underestimate the underlying arboviral disease burden. In Benin, a country with a tropical wet-dry climate, the serological evidence of DENV infections in travellers returning from Benin have been described [2]. To gain data about the occurrence of arboviruses, a longitudinal arbovirus surveillance study was conducted.

Mosquitoes were collected from June 2020 until October 2021 at five sites in two bioclimatic regions in Benin (Additional file 1: Fig. S1). Specimens were morphologically identified on chilled tables and samples were tested with pan-*Flavivirus*, pan-*Alphavirus* and pan-*Orthobunyavirus* reverse transcription-polymerase chain reaction (RT-PCR) with primers shown in the Additional file 1: Table S1. The amplicons were sent for sequencing (LGC Genomics, Berlin, Germany) and sequences were analysed with Geneious v9.1.7 (Biomatters, Auckland, New Zealand).

A total of 3749 female mosquitoes, including two genera and four species, were captured during the sampling period (Additional file 1: Table S2). The dominant species was *Aedes aegypti* with 2970 specimens, followed by *Culex quinquefasciatus* with 753 specimens. DENV-3 specific RNA was detected in one sample (pool of 25 *Aedes aegypti*) collected with human landing catch (HLC) on 7th July 2021 in Porto Novo. Phylogenetic analysis revealed that the DENV-3 strain from Benin is grouped into the genotype III clade. The sequence clusters in a distinct clade within a monophyletic Africa clade (Fig. 1). Sequence analysis showed a close relationship to DENV-3 strains from humans in Burkina Faso and Ethiopia with a paired identity at the nucleotide level of 98.8% and 98.5%, respectively.

**Fig. 1:**
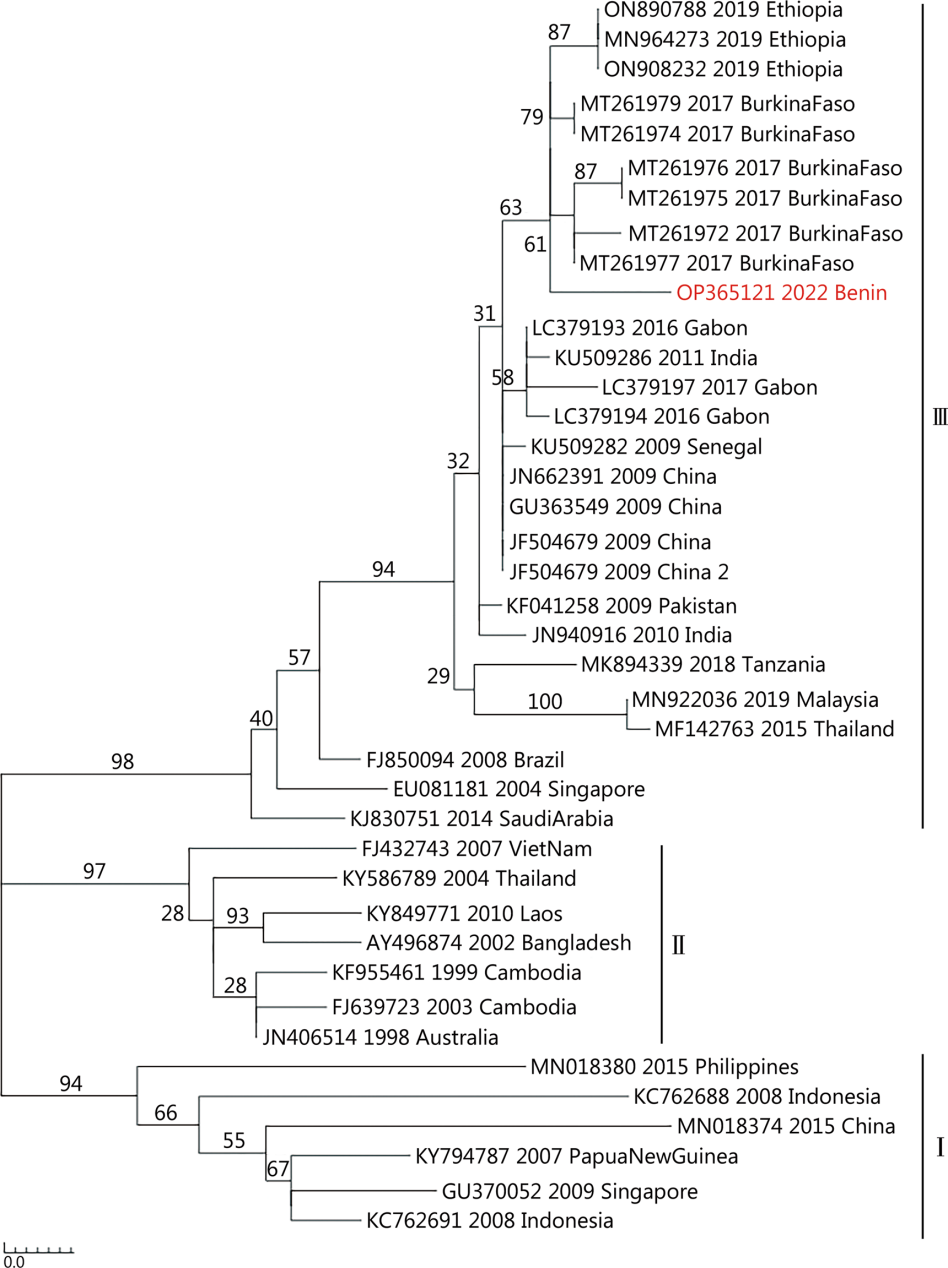
Phylogenetic tree of detected dengue virus serotype 3. The genetic analysis is based on a 483-nt sequence alignment comprising the C-prM gene sequences of DENV serotypes and reconstructed using Maximum Likelihood method and a General Time Reversible model implemented by using MEGA3 software (www.megasoftware.net). Values at nodes indicate bootstrap support (1000 replicates). Roman numerals denote the different genotypes of DENV-3. The sequence from Benin was gained using the primers D1 and D2 (Additional file 1: Table S1). The analysis involved 40 nucleotide sequences and GenBank accession numbers are provided for reference genomes. DENV dengue virus

We report the first detection of DENV in mosquitoes in Benin, giving another indication that this virus is endemically occurring in this region. Phylogenetic analysis showed a close relationship to DENV strains from Burkina Faso and Ethiopia. Burkina Faso experienced a greater DENV outbreak in 2016 and 2017. The detection of DENV-3 genotype III strain in Benin and surrounding countries supports the suggestion that there is an ongoing circulation of different DENV strains in the West African countries. Specifically, DENV-3, has become increasingly common in West and Central Africa in the last decade and has been responsible for several outbreaks in Senegal (DENV-3 accounting for 65% of all gained sequences) [3], Burkina Faso [4] and Gabon [5]. Until now, Benin did not report any outbreak of DENV, but it must be assumed that human infections occur regularly. Due to the restricted availability of diagnostic capacity, most investigations are performed only during clearly noticeable outbreak periods. Our study highlights that there is a need to implement further investigations and surveillance strategies to prevent and control future outbreaks of mosquito-borne viruses in Western Africa.

## Supplementary Information

**Additional file 1: Fig. S1.** Map of Benin with trapping sites and number of collected mosquitoes. **Table S1.** List of the primer sequences used for reverse transcription polymerase chain reaction. **Table S2.** Total number of collected female mosquitoes.

## Data Availability

Not applicable.

## Abbreviations

DENV: Dengue virus
HLC: Human landing catches
RNA: Ribonucleic acid
RT-PCR: Reverse transcription polymerase chain reaction
