# Supplementary material for: Surveillance of arthropod-borne viruses in Benin, West Africa 2020–2021: detection of dengue virus 3 in Aedes aegypti (Diptera: Culicidae)
Source: Mil Med Res. 2022 Nov 14;9:64. doi: 10.1186/s40779-022-00425-9 (PMC9661747; doi:10.1186/s40779-022-00425-9)
Supplement: Supplementary file 1 — Additional file 1: Fig. S1. Map of Benin with trapping sites and number of collected mosquitoes. Table S1. List of the primer sequences used for reverse transcription polymerase chain reaction. Table S2. Total number of collected female mosquitoes. [file 40779_2022_425_MOESM1_ESM.pdf]

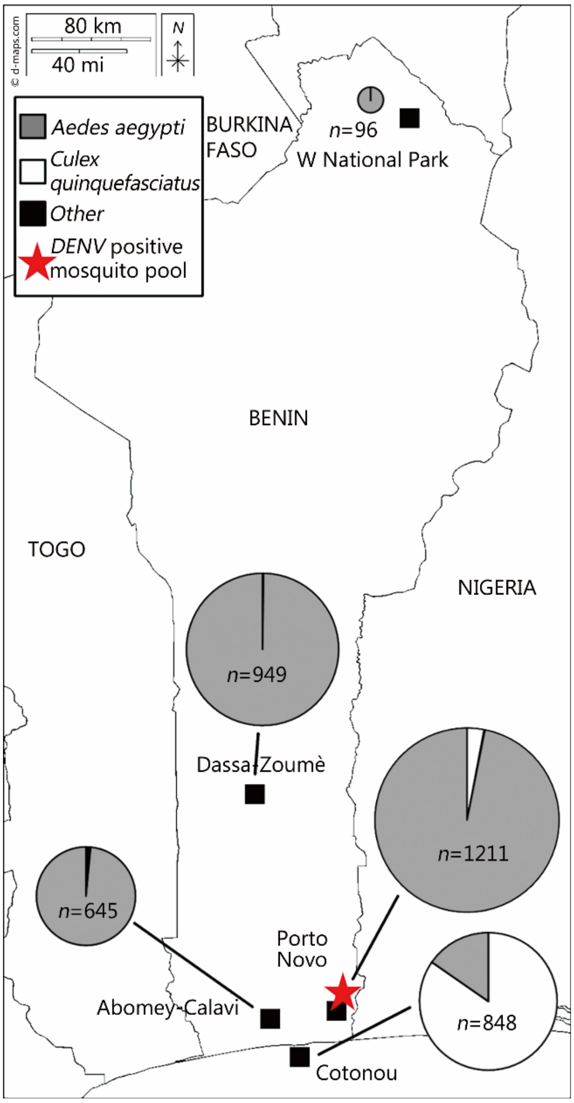

Fig. S1 Map of Benin with trapping sites and number of collected mosquitoes. Mosquitoes for arbovirus screening were sampled at four sites in the Guinean zone [Abomey-Calavi (6.418736°N, 2.3425287°E), Cotonou (6.364528°N, 2.441564°E), Dassa-Zoumè (7.783625°N, 2.185264°E), Porto Novo (6.510439°N, 2.604147°E)] and one site in the Sudanian zone [W National Park (12.040653°, 3.034178°)]. For virus screening, up to 25 mosquitoes were pooled in a 2 ml reaction tube according to the trapping site, date, and species. The pools were titrated with 500 µl of cell culture medium [high-glucose Dulbecco's modified Eagle's medium (DMEM; Sigma-Aldrich, St.Louis, MO)] and zirconia beads (2 mm, Carl Roth, Karlsruhe, Germany). Mosquitoes were homogenized for 4 min on a vortexer. The suspension was clarified by centrifugation for 1 min at 8000 r/min and 4 °C and RNA was extracted with a QIAamp viral RNA mini kit according to the manufacturer's protocol (Qiagen, Hilden, Germany). DENV dengue virus

Table S1 List of the primer sequences used for reverse transcription polymerase chain reaction

Target	Primer name	Primer sequence (5' - 3')	Reference
Pan-*Flavivirus*	Forward (mFU1)	TACAACATGATGGGAAAGCGAGAGAAAAA	[1]
	Reverse (CFD2)	GTGTCCCAGCCGGCGGTGTCATCAGC	
Pan-*Alphavirus*	Forward (VIR2052)	TGGCGCTATGATGAAATCTGGAATGTT	[2]
	Reverse (VIR2052)	TACGATGTTGTCGTCGCCGATGAA	
Pan-*Orthobunyavirus*	Forward (Cal/Bwa)	GCAAATGGATTTGATCCTGATGCAG	[3]
	Reverse (Cal/Bwa)	TTGTTCCTGTTTGCTGGAAAATGAT	
	Forward (Bun)	CTGCTAACACCAGCAGTACTTTTGAC	
	Reverse (Bun)	TGGAGGGTAAGACCATCGTCAGGAACTG	
	Forward (Wyeomyia)	ATGTCTGAAATTGTATTTGATGATATTGG	
	Reverse (Wyeomyia)	TATTTCGATTCCCCGGAAAGT	
	Forward (Oropouche)	GGCCCATGGTTGACCTTACTTT	
	Reverse (Oropouche)	ACCAAAGGGAAGAAAGTGAAT	
Dengue virus C-prM gene	Forward (D1)	TCAATATGCTGAAACGCGCGAGAAACCG	[4]
	Reverse (D2)	TTGCACCAACAGTCAATGTCTTCAGGTTC	

Table S2 Total number of collected female mosquitoes

Sampling date	Sampling site	Sampling method	*Aedes aegypti* (unengorged/blood-fed)	*Aedes albopictus*	*Aedes vittatus*	*Culex quinquefasciatus* (unengorged/blood-fed)	Not identified
01.06.2020	Abomey-Calavi	BGS	65/3				1
01.07.2020	Abomey-Calavi	BGS	0/6				
02.07.2020	Porto Novo	BGS	233/25				
02.07.2020	Porto Novo	GAT	161/9				
02.07.2020	Porto Novo	HLC	249/32				
01.10.2020	Cotonou	BGS	71/3				
01.10.2020	Cotonou	GAT	11/10				
05.10.2020	Dassa-Zoumè	GAT	5/9				
05.10.2020	Dassa-Zoumè	BGS	32/11				2
01.03.2021	Cotonou	GAT	4/4			3/1	
02.03.2021	Cotonou	GAT	0/2			6	
02.03.2021	Cotonou	BGS				160/3	2
03.03.2021	Cotonou	BGS	9			270/5	
03.03.2021	Cotonou	GAT	1/1				
04.03.2021	Cotonou	BGS	4			106/4	
05.03.2021	Cotonou	BGS	4/4			150/5	
05.03.2021	Cotonou	GAT	1			3	
23.03.2021	Cotonou	GAT	0/1				
18.05.2021	Abomey-Calavi	HLC	117/10	1	1		1
09.06.2021	Abomey-Calavi	BG	26				
09.06.2021	Abomey-Calavi	HLC	269				
24.06.2021	Porto Novo	HLC	111/10				

28.06.2021	Dassa-Zoumè	HLC	0/5				
07.07.2021	Abomey-Calavi	HLC	99/36	8	2		
07.07.2021	Porto Novo	HLC	256	2			3
29.07.2021	Dassa-Zoumè	HLC	101/16				
30.07.2021	Dassa-Zoumè	HLC	33				
01.08.2021	Dassa-Zoumè	HLC	134/56			0/1	
25.08.2021	Porto Novo	HLC	73/8			5/1	
26.08.2021	Porto Novo	BGS	3			30	
01.09.2021	Dassa-Zoumè	HLC	100				
02.09.2021	Dassa-Zoumè	HLC	134	1			
02.09.2021	Dassa-Zoumè	BGS	30				2
04.09.2021	Dassa-Zoumè	HLC	77				
05.09.2021	Dassa-Zoumè	HLC	200				
17.10.2021	W National Park	HLC	71/25				
Subtotal (% of total)			2684/286 (71.6/7.6)	12 (0.3)	3 (0.1)	733/20 (19.6/0.5)	11 (0.3)
Total			3749				

BGS (Biogents, Regensburg, Germany) were equipped with BG-Lure (Biogents, Regensburg, Germany) as an attractant and were set in the afternoon for 24 h, passive GAT (Biogents, Regensburg, Germany) were filled with rain water, placed on the ground in a shaded area, and mosquitoes were captured for 24 h, and HLC was performed during dusk and dawn for 2 h, respectively. For HLC World Health Organization guidelines and protocols were strictly followed, and ethical concerns have been raised and evaluated. *HLC* human landing catches, *BGS* BG-Sentinel mosquito trap, *GAT* Gravid Aedes Trap

1. Chao DY, Davis BS, Chang GJJ. Development of multiplex real-time reverse transcriptase PCR assays for detecting eight medically important flaviviruses in mosquitoes. J Clin Microbiol. 2007;45(2):584-9.
2. Eshoo MW, Whitehouse CA, Zoll ST, Massire C, Pennella TTD, Blyn LB, et al. Direct broad-range detection of alphaviruses in mosquito extracts. Virology. 2007;368(2):286-95.
3. Lambert AJ, Lanciotti RS. Consensus amplification and novel multiplex sequencing method for S segment species identification of 47 viruses of the *Orthobunyavirus*, *Phlebovirus*, and *Nairovirus* genera of the family Bunyaviridae. J Clin Microbiol. 2009;47(8):2398-404.
4. Lanciotti RS, Calisher CH, Gubler DJ, Chang GJ, Vorndam AV. Rapid detection and typing of dengue viruses from clinical samples by using reverse transcriptase-polymerase chain reaction. J Clin Microbiol. 1992;30(3):545-51.
